# A rare case of crossed pulmonary arteries in an infant - case report

**DOI:** 10.1186/1749-8090-8-79

**Published:** 2013-04-11

**Authors:** Jin Chen, Yue Feng

**Affiliations:** 1 Intensive Care Unit, Zhejiang Hospital, 12 Lingyin Road, Hangzhou, 310013, China, P.R; 2 Department of Radiology, Zhejiang Hospital, 12 Lingyin Road, Hangzhou, 310013, China, P.R

**Keywords:** Anomalies of pulmonary arteries, Malposition of pulmonary arteries, Computed tomography

## Abstract

Crossed pulmonary arteries are a quite rare form of pulmonary arterial malposition. In this anomaly, the left pulmonary artery originates from the pulmonary trunk to the right and usually above the origin of the right pulmonary artery. Both pulmonary arteries cross each other on their course to each respective lung. We presented a case of a Chinese infant with crossed pulmonary arteries. Physical examination showed a mild cyanosis and continuous machine-like heart murmur in the 2 intercostal space at the left sternal border. An echocardiogram revealed pulmonary hypertension, atrial septal defect, patent ductus arteriosus and ostial stenosis in the inferior left pulmonary vein. Dual-source CT angiography was performed for further evaluation of pulmonary trunk and its branches. Dual-source CT angiography showed origin of left pulmonary artery from the pulmonary trunk in a plane superior to that of the right pulmonary artery. The branch pulmonary arteries then crisscrossed as they coursed to their respective lungs. In summary, we report an infant with crossed pulmonary arteries who was diagnosed during dual-source CT angiography. Three-dimensional reconstruction is useful for visualizing this condition. Knowledge of this rare anomaly will help in the differential diagnosis of pulmonary artery abnormalities.

## Background

Crossed pulmonary arteries are a quite rare form of pulmonary arterial malposition, usually found in association with congenital cardiac and extracardiac diseases. So the recognition of this anomaly is seriously important. In this anomaly, the left pulmonary artery (LPA) originates from the pulmonary trunk to the right and usually above the origin of the right pulmonary artery (RPA) [1]. Both pulmonary arteries cross each other on their course to each respective lung [2].

## Case presentation

**Figure 1:**
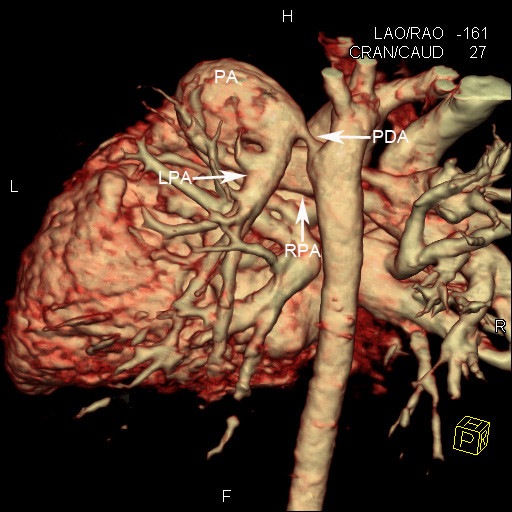
**Volume-rendered reformation.** The origin of left pulmonary artery (LPA) from the pulmonary trunk (PA) in a plane superior to that of the right pulmonary artery (RPA). PDA is also seen.

**Figure 2:**
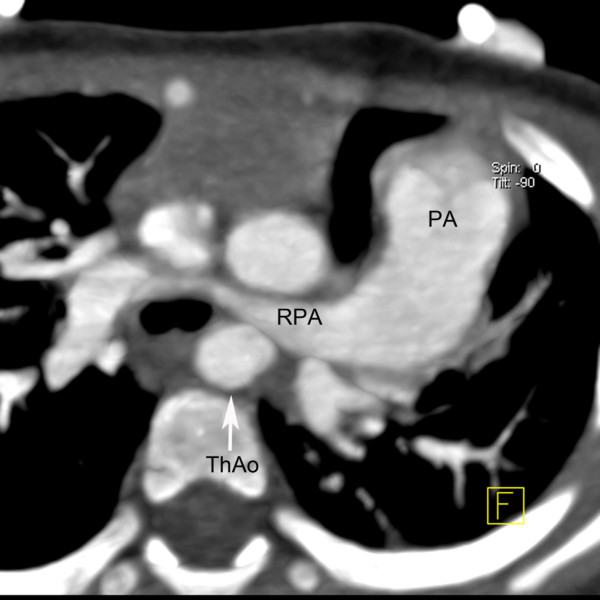
**Multiplanar reconstruction.** The right pulmonary artery (RPA) coursed to the right lung. Thoracic aorta (ThAo) located anterior to the thoracic vertebrae.

**Figure 3:**
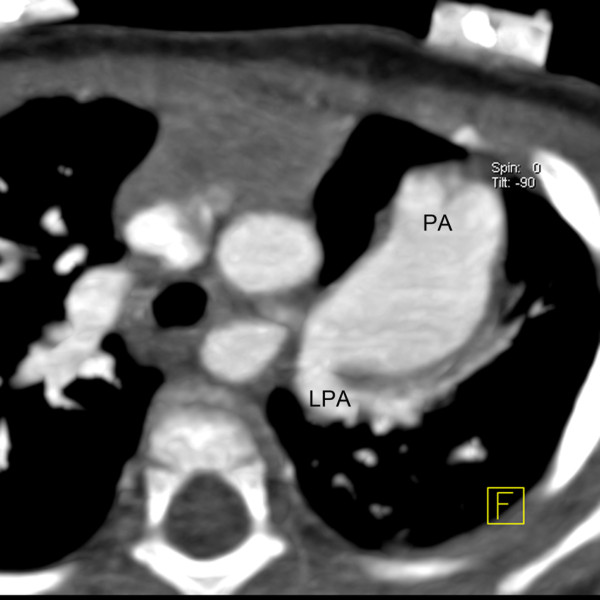
**Multiplanar reconstruction.** The origin of left pulmonary artery (LPA) from the pulmonary trunk (PA).

**Figure 4:**
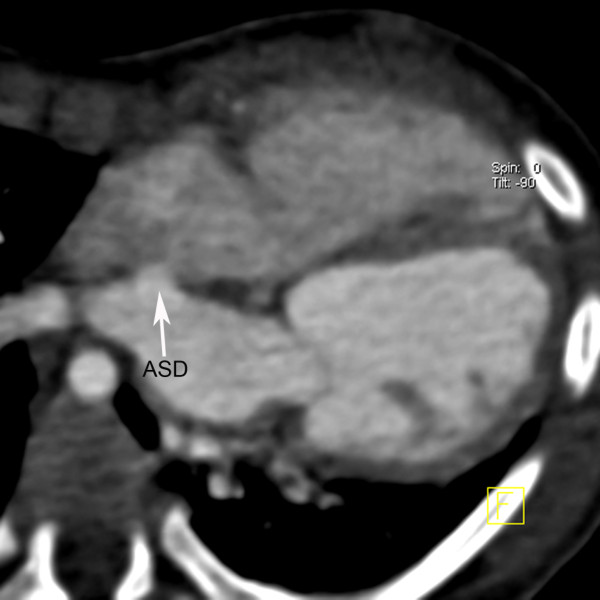
**Multiplanar reconstruction.** Atrial septal defect (ASD) is seen.

**Figure 5:**
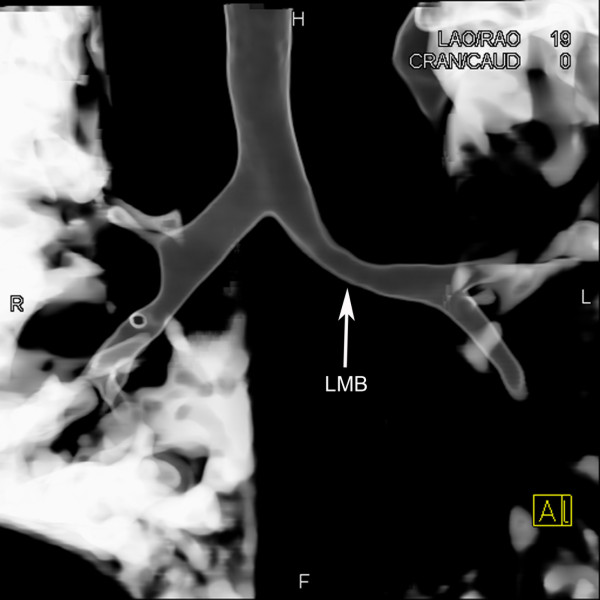
**Volume-rendered reformation.** Stenosis of the left main bronchus (LMB).

A 10-month-old Chinese boy was referred to our hospital for heart murmur. Physical examination showed a mild cyanosis and continuous machine-like heart murmur in the 2 intercostal space at the left sternal border. An echocardiogram revealed pulmonary hypertension, atrial septal defect (ASD), patent ductus arteriosus (PDA) and ostial stenosis in the inferior left pulmonary vein. But the echocardiography could not demonstrate the bifurcation of pulmonary arteries clearly. Dual-source CT (Somatom Definition, Siemens Medical Systems, Forchheim, Germany) angiography was performed for further evaluation of pulmonary trunk and its branches. Dual-source CT angiography showed origin of LPA from the pulmonary trunk in a plane superior to that of the RPA (Figure 1). The branch pulmonary arteries then crisscrossed as they coursed to their respective lungs (Figures 2 and 3). The fusion of contrast between the aorta and pulmonary trunk through a communication was demonstrated by CT images (Figure 1). CT images also showed ASD (Figure 4). Thoracic aorta located anterior to the thoracic vertebrae and left main bronchus was oppressed (Figures 4 and 5). The origin and course of the coronary arteries were normal.

## Discussion

Malposition of the pulmonary arteries is a rare congenital heart disease with two forms [3]. Crossed pulmonary arteries are the classic form of malposition of the branch pulmonary arteries. But there are limited reports about crossed pulmonary arteries. According to our search of the literature, fewer than 39 cases had been reported up to 2013. The first description of crossed pulmonary arteries was reported by Jue et al. in 1966 [4]. The etiology of crossed pulmonary arteries has not been established. This form of malposition might be attributed to the differential growth within the pulmonary trunk, resulting in counterclockwise rotation of the normal origins of the branch pulmonary arteries [1,4,5]. Some patients also had other congenital and chromosomal abnormalities, including trisomy 18 and 22q11 deletions [4].

In patients with crossed pulmonary arteries, the origin of the LPA from the pulmonary trunk usually lies above the RPA. From these abnormal positions, the pulmonary arteries cross each other as they proceed to their respective lungs. Crossed pulmonary arteries may be associated with other cardiac anomalies, including truncus arteriosus, interrupted aortic arch, tetralogy of Fallot, ASD and left superior vena cava [5-7]. Associated PDA and ASD were discovered in our patient. Despite the abnormal location and course of the branch pulmonary arteries, crossed pulmonary arteries do not cause hemodynamic abnormalities. When pulmonary pressure supersedes the systemic pressure, blood will shunt right to left across existing cardiovascular channels, such as the ASD or PDA, and result in intractable systemic hypoxaemia and cyanosis [8].

Airway obstruction is well-recognized in children with congenital heart disease [9]. It may be related to extrinsic compression by vascular structures such as dilated branch pulmonary arteries, the malpositioned descending aorta and the pulmonary arterial sling. Thoracic aorta located anterior to the thoracic vertebrae in our patient. The left main bronchus was oppressed by the malpositioned thoracic aorta.

Becker et al. also reported a “lesser form” of malposition of the pulmonary arteries in 1970 [6]. In this form, the left pulmonary artery ostium lies directly superior to the ostium of the right pulmonary artery, but the branches are not crossed [3].

Cardiac angiography, echocardiography, magnetic resonance imaging and computed tomography were all used to diagnose crossed pulmonary arteries [1,5,10,11]. The unusual origin and course of the branch pulmonary arteries complicate accurate interpretation of the location of the catheter and the relationship of the vessels during angiography [11]. The bifurcation of pulmonary arteries may not be clearly demonstrated by echocardiography, so diagnosis of this lesion is still difficult. Magnetic resonance imaging has the advantages of multiplanar views, but long acquisition time limits its use in patients who are in an unstable condition. Due to high spatial resolution, short scan time and the resultant reduced requirement for sedation or general anaesthesia, dual-source CT angiography is useful for identifying cardiac anomalies [12,13]. Three-dimensional reconstruction may improve the understanding of this anomaly. It also permits preoperative simulation and postoperative assessment. The use of ionizing radiation and iodinated contrast agents are drawbacks of CT. These risks can be reduced by using optimal CT techniques, such as low-dose CT protocols, tube current modulation, saline flush, and a dual power injector [14-16].

Crossed pulmonary arteries must be distinguished from the pulmonary arterial sling. In a pulmonary sling, the left pulmonary artery originates from the right pulmonary artery and courses between the esophagus and the trachea as it passes from the right hilum to the left lung [5]. It might cause airway obstruction.

Crossed pulmonary arteries usually do not require surgical correction, unless they are associated with stenosis or other lesions. However, in patients who have unrepaired systemic to pulmonary communications with associated cyanosis, surgical intervention can be fatal [17]. So the patient did not undergo surgery in our institution.

## Conclusions

In summary, we report an infant with crossed pulmonary arteries who was diagnosed during dual-source CT angiography. Three-dimensional reconstruction is useful for visualizing this condition. Diagnostic key point of crossed pulmonary arteries is to reveal the true relationship between the LPA and RPA. Knowledge of this rare anomaly will help in the differential diagnosis of pulmonary artery abnormalities.

## Consent

Written informed consent was obtained from parents of the patient for publication of this case report and any accompanying images. A copy of the written consent is available for review by the Editor-in-Chief of this journal.

## Abbreviations

LPA: The left pulmonary artery; RPA: The right pulmonary artery; ASD: Atrial septal defect; PDA: Patent ductus arteriosus; ThAo: Thoracic aorta; PA: The pulmonary trunk; LMB: The left main bronchus.

## Competing interests

The authors declare that they have no competing interests.

## Authors’ contributions

JC was the treating physician and was responsible for the treatment; YF was the radiologist responsible for CT imaging. Both authors read and approved the final form manuscript.
